# Proceedings of essential health care package development, in Botswana and Sierra Leone, November 2022

**DOI:** 10.1186/s12919-025-00322-8

**Published:** 2025-03-21

**Authors:** Humphrey Cyprian Karamagi, Solyana Ngusbrhan Kidane, Pierre Claver Kariyo, Araia Berhane Mesfin, Isabella Maina, Gertrude Avortri, Hyppolite Kalambay Ntembwa

**Affiliations:** 1 Data Analytics and Knowledge Management, World Health Organization Regional Office for Africa, Brazzaville, Republic of Congo; 2 Integrated Service Delivery for West Africa, Assistant Regional Director, World Health Organization Regional Office for Africa, Ouagadougou, Burkina Faso; 3 Communicable Disease, Ministry of Health, Asmara, Eritrea; 4 Ministry of Health, Nairobi, Kenya; 5 Integrated Service Delivery for East and Southern Africa, Assistant Regional Director, World Health Organization Regional Office for Africa, Harare, Zimbabwe; 6 Service Delivery System, Assistant Regional Director, World Health Organization Regional Office for Africa, Brazzaville, Congo

**Keywords:** Essential health care package, Essential health services package, Service delivery, Botswana, Sierra Leone, UHC, Interventions

## Abstract

The development of essential health care package (EHCP) has been recognized as a critical tool for guiding country level actions towards Universal Health Coverage (UHC). Although countries’ packages vary in scope, many utilize the package to guide resource allocation, equity, advocacy, prioritization of services, political commitment, and accountability. The concept of health packages has evolved from basic packages (focusing on limited high-burden conditions), to benefit- (cost-effective interventions) and essential packages (what people need, with benefits as sub-packages). The purpose of this proceeding was to document processes from Botswana and Sierra Leone workshops, which aimed to support country conceptualization of an EHCP, including content and scope. More specifically, the workshop aimed to gain consensus on identification of conditions to be addressed in each age cohort, rationalizing the EHCP interventions across public health functions, levels of care and age cohorts. Technical working groups were constructed for each age cohort and tasked to lead the appraisal of interventions for technical comprehensiveness, contextualization to country needs, and mapping of interventions to the appropriate levels of care. As a result, the countries' draft EHCPs were developed, encompassing interventions for 80 + conditions. The EHCP is expected to set precedence in defining ‘essential’ interventions for the population, structurally promoting integration of health services, and providing succinct guidance to partners, and stakeholders on the country's priorities, in terms of health interventions to be delivered at various levels. Many countries are striving to re-pivot their health systems, in order to meet the evolving contextual needs of the population and ensure their systems remain fit for purpose. EHCPs can be utilized to guide health sector inputs, for robust system functionality and attainment of UHC.

## Background

Universal health coverage (UHC) is a high priority for countries in the World Health Organization African (WHO AFRO) region. Although UHC remains a long-term goal, its attainment requires innovative and reformed approaches in service delivery systems. The integrated nature of Sustainable Development Goals (SDGs) as well as UHC necessitate multiple stakeholder engagement and synergy, for delivery of essential services demanded by the population. Defining UHC for an individual country remains largely varied, with different countries embarking on various approaches and conceptualizations of UHC. In order to provide succinct guidance, the WHO AFRO regional committee in 2018 adopted the regional framework for health system development [1] and later, a regional framework for provision of essential health services through strengthened district/local health systems adopted in 2019 [2]. These frameworks provide guidance to countries on the realignment of investments needed to attain a comprehensive set of health and health-related outcomes. It also provides linkages between health system investments and health service outcomes, to ensure synergies of action across the health system results chain framework.

The development of essential health care package (EHCP) has been recognized as critical tool for UHC in recent policy discourse. An EHCP is defined as “a list of public health, clinical services and interventions, that a country has determined as priority for its population”. It outlines the comprehensive list of essential health services and interventions that the government is providing or aspiring to provide for its citizen [3]. EHCPs are considered a useful tool by governments to articulate the set of interventions they aspire to deliver, which can be used to inform health investment priorities [4]. Although respective countries’ EHCPs vary in scope and breadth of interventions, countries utilize the EHCP to guide resource allocation, equity, advocacy, prioritization of services, political commitment, and accountability [4, 5]. EHCPs consider a heath action received by a person or family (eg. pentavalent vaccination), as an intervention; whereas a set of interventions delivered together (e.g. childhood immunizations) is coined as a service. As such, the EHCP is used to describe a package of health interventions organized by age cohort, levels of care and public health function.

The concept of EHCPs has evolved from basic packages (focusing on limited high burden conditions), to benefit (cost-effective interventions) and essential packages (what people need, with benefits as sub-packages) [6–8]. Basic packages traditionally focus on certain age groups (eg. Mother and child focus), primary and to some extent secondary levels of care, mainly curative services, and donor-funded, which tend to cease once the funding halts. During the Millennium Development Goals (MDGs) era, for instance, resources from developmental assistance heavily focused on Reproductive Maternal Neonatal Child and Adolescent Health (RMNCAH) and infectious diseases. This was indicative of the priority focus on high-burden conditions at the time, which sidelined the focus needed for non-communicable diseases and injuries. Given the limited resources and the necessity to prioritize interventions, countries shifted focus to cost-effective packages as the core milestone to guide their delivery systems. Although practically appealing, the cost–benefit packages in isolation are unable to accommodate all interventions of critical importance to an individual.

The aforementioned issues are addressed through the EHCPs, as the package is primarily defined based on the needs of the population and subsequently used to guide the development of benefit packages, that aim to incrementally avail the needed service. The EHCP principles also echo strategic shifts that are aligned with the multi-sectorial and inclusive nature of SDGs, in contrast to basic packages. With the essential health care package, a shift in emphasis is placed on all essential services, with models of services for different populations defined, across all age cohorts (child to elderly) and public health functions, accommodating for planned and emergency services, focusing on sectors that influence health, and moving towards government funded services. It is against this background that the proceeding aims to share insights from countries on the EHCP development and outline emerging issues for health system. The purpose of the Botswana and Sierra Leone workshops were to support country conceptualization of an EHCP, including content and scope. Furthermore, the sessions aimed to provide technical coordination and development of EHCP, in a stepwise manner, through national teams’ stewardship.

## Methodology

### Actions taken prior to the meeting

The concept and approach of UHC has informed WHO AFRO development of essential health care package (EHCP) – a country toolkit for action, providing stepwise approach [8].The package outlines the key services that a country will work towards, defined for all people at all ages, at all levels of care. The EHCP is implemented in a phased manner, through benefit packages that the country can afford at a given time. An EHCP is central to the health sector, becoming the key anchor around which, all investments and results coalesce.

### EHCP toolkit

The EHCP development toolkit is accompanied by the compendium of intervention. The AFRO essential health services toolkit [9] is an open compendium of interventions available within the integrated African Health Observatory (iAHO). Interventions are defined here as ‘activities or actions provided for an individual to improve the health condition, by preventing infections or diseases, cure diseases or suppressing disease-causing germs/viruses or reducing the severity or duration of an existing disease, or by restoring function lost due to diseases or injuries’ [10]. The tool contains a menu of interventions defined by (1) age cohort (pregnancy/newborn, children under 5 years, late childhood, adolescence, adulthood, elderly), (2) public health functions (health promotion, disease prevention, curative services, rehabilitative services, palliative care), (3) health conditions (infectious, non-communicable, trauma/injury), and (4); and level of care (community services, primary care, secondary hospital, tertiary hospital).

The EHCP development toolkit within the WHO AFRO region was initially implemented in Eritrea and Lesotho. The toolkit assisted these two countries in a stepwise approach of EHCP development, whilst unpacking the practical implications of systems framework for UHC. For instance, the countries were able to systematically identify the necessary investments required across the levels of care. Furthermore, aspirations and future direction of the EHCP were used to guide the health sector policy and strategic plans, on how new services would be introduced, coverage upscaled and systems designed to protect financial burden when accessing health care.

The experiences and lessons learnt in countries were used to refine EHCP development toolkit. For instance, the burden of disease data was expanded to include local data and information beyond the open sources used, as countries expressed the necessity to account for niche peculiarities that may prevail. Targeted appraisal processes from specialized centers, associations and community representatives were strongly recommended by countries, and refined in the process of EHCP development. In addition, both Eritrea and Lesotho convened extensive technical engagements with disease and health programs and other key players in their respective Ministries of Health (MoH). The process was reported as a catalyst to program integration initiatives.

More recently, Botswana and Sierra Leone embarked on the process to define their countries’ EHCPs. The EHCPs development and subsequent implementation in both countries are highly prioritized by their respective governments and viewed as a critical milestone in setting the agenda. The Botswana national health policy, under the theme “Towards a healthier Botswana”, has as its vision, achieving and maintaining highest level of health and well-being*.* The vision of Sierra Leone national health policy states that ‘All people should have access to affordable quality healthcare services and health security without suffering undue financial hardship’. This vision is stated in the Sierra Leone 2021–25 National Health Sector Strategic Plan. Central to this goal is the revision and development of an EHCP for the country.

### Development process in Botswana and Sierra Leone

#### Desk review

Prior to the workshop, a desk review was undertaken in Botswana and Sierra Leone. The review focused on relevant documents and materials pertaining to health services provision in general, and available health care packages, to better understand the current situation and context of the respective countries, as well as priorities set by their Ministries of health, for harmonization and complementarity. The relevant documents included National Health policies; National Health strategies; comprehensive recent reviews of the health sector strategies; and previous national health packages. The review generated information on the status of the health sector and provided insights on the requirements going forward. The documents provided critical information on the existing burden of disease in the country as well as progress made in the implementation of the EHCP and the challenges thereof. Visits to health facilities were also used as avenues to shed light on the status quo of service delivery within a country.

#### Visit to health facilities

In both countries, randomly selected health facilities located in both urban and rural areas, representing different levels of care were visited. The visit gave an insight to the current context of the health services, especially the standardized practices, types of health personnel, specialized equipment used in delivering interventions.

#### Conduct of the workshop

The workshop in Botswana was held from 24 October to 4 November and in Sierra Leone, 7- 18 November 2022.

The technical meetings aimed to gain common understanding of the essential package conceptualization and process, covering conditions to be addressed in each age cohort, rationalizing the essential health care package interventions across public health functions, levels of care and age cohorts.

Participants drawn from different areas of expertise, disease programs and specialties attended the workshops in Sierra Leone and Botswana. In Sierra Leone, the workshop was held in Tokeh, with 50 senior participants affiliated with various disease programs and organizations, such as communicable disease, non-communicable, injuries, health economy, public health, primary health care, Non-government organizations (NGOs), United Nations (UN) agencies, Laboratory services, specialized hospitals, Human resources management, district management, Reproductive and maternal health, food and nutrition, rehabilitative and palliative care. In Botswana, the workshop was held in Gaborone, with a total of 66 participants drawn from the Ministry of health (deputy principal secretaries; deputy director of health services and departmental heads, directors, and program officers) the districts including district health management team (DHMT) coordinators; the hospitals especially Princess Marina Hospital (PMH); partners such as United State Agency for International Development (USAID); government agencies eg Statistics Botswana, insurance agencies (e.g. Botswana Medical Aid) and Academia including University of Botswana among others.

#### Technical working groups

The two countries constructed Technical Working Groups (TWG) (Table 1) based on the members' specialty and programme expertise. Each team had a chair and secretariate, who facilitated the discussion and documented agreed changes. TWG then received technical support from external experts.

**Table 1:** Technical working groups

Country	Technical working groups
Botswana	• Pregnancy and New-born (up to 28 days); • Early Childhood (29 days – 59 months); • Late Childhood (6 −13 Years); • Adolescent and Youth (14 – 29 Years); • Adulthood (30 – 64 Years) and • Elderly (65 + Years)
Sierra Leone	• Pregnancy/reproductive health • Childhood (under 5 years) • Late childhood and adolescent (5–19) • Adults (20–64) • Elderly (65 +)

#### EHCP Development process

The EHCP development encompassed stepwise process, grouped under the following five steps.

- Step 1—The conditions to be addressed by the essential health care package was primarily based on evidence of mortality, morbidity, and risk factors as well as areas of key concerns, such as those targeted for elimination, control, those localized and of key concern to certain regions in the country and those of public health importance. Stakeholders utilized burden of diseases estimates, complimented with local data and information where available, to decipher the conditions to be addressed in the EHCP.
- Step 2—Interventions were subsequently extracted from the WHO compendium for selected conditions. For each age cohort, interventions were defined by the public health function (health promotion, prevention, curative, rehabilitative, palliative care) and levels of care (community, primary, secondary, and tertiary).
- Step 3 – Rationalization of the EHCP was undertaken, through extensive review and consultations within the TWGs. For selected conditions in each age cohort, interventions were identified across the public heath functions; Health Promotion – cross sectoral risk factor mitigation; Disease Prevention – health sector actions; Curative – clinical interventions; Rehabilitative – restorative of function post episode and Palliative – easing of discomfort. Furthermore, the interventions were mapped to where they are most effectively delivered.
- Step 4 – Harmonization of interventions was undertaken through regular review and engagement of TWGs. Plenary was held daily for TWGs to present succinct approaches and sample of interventions reviewed for each of the age cohorts. Deliberations were made in plenary and consensus reached on matters, for cohesion and comprehensiveness of standard services across the life course.
- Step 5 – Recognizing the scope and impact of EHCPs in countries, meticulous appraisals were conducted with stakeholders. The first level of appraisal is by the TWG coordinating the package, to ensure harmonization, scope and language, followed by MOH Technical programs (second level), to ensure interventions are reflected appropriately (language, scope, etc.); and public, and government bodies, development partners, academia) for validation (third level).

**Fig. 1:**
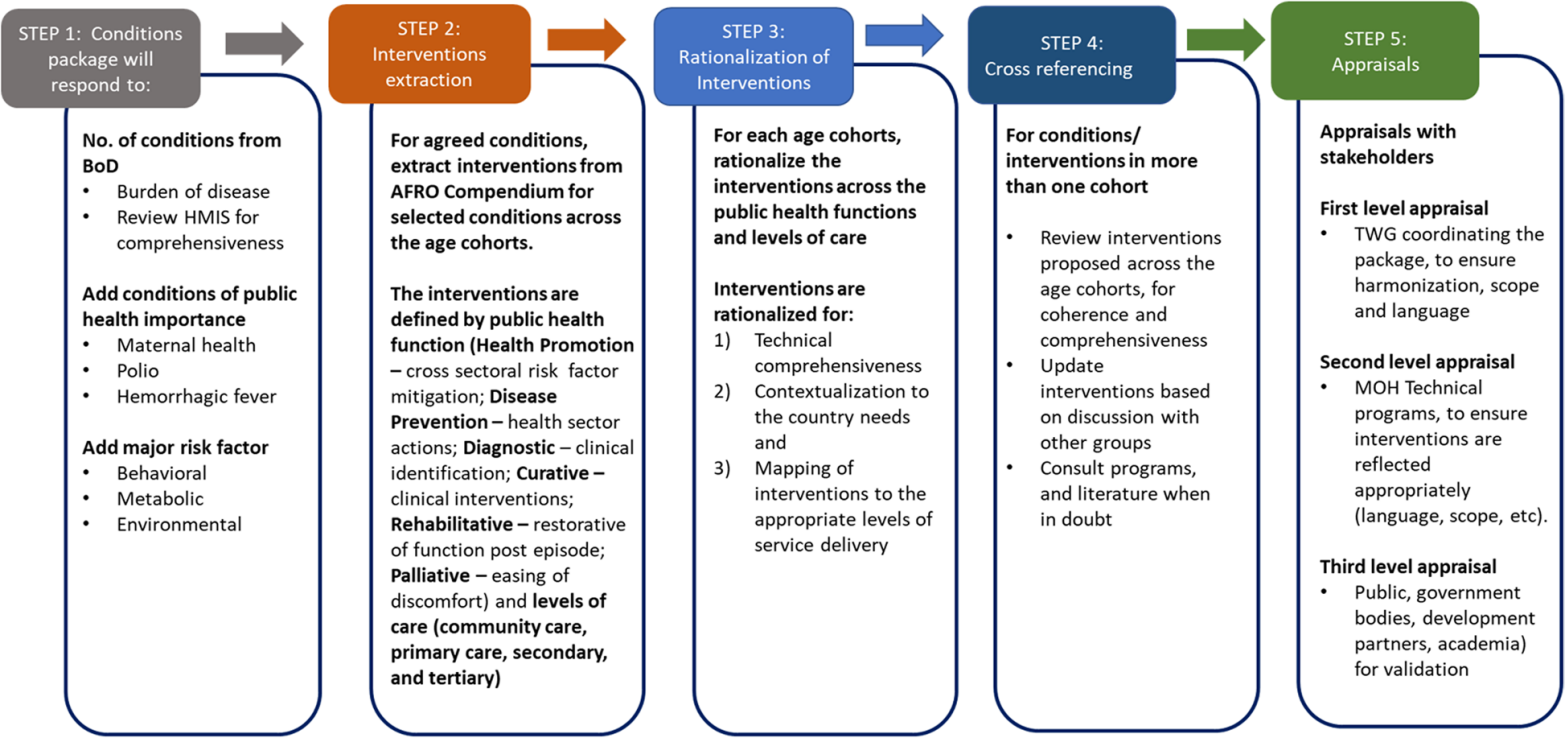
Essential health care package development

At the start of the workshops, presentations were made on critical areas covering the background and rationale of the EHCP development, encompassing steps outlined above (Fig. 1).

## Results

### Condition selection

The conditions to be addressed by the EHCP were primarily based on evidence of mortality, morbidity, and risk factors as well as areas of key concerns, such as those targeted for elimination, control, of public health importance etc. Burden of disease (BoD) data [11] was generated for each age cohort and reviewed extensively for consensus. Initially, the BoD data had more than 50 conditions enlisted for each cohort in Sierra Leone and Botswana. This was reviewed by MOH directors and program managers, partners, NGOs and wider stakeholders prior to the workshop.

On the first session of the workshop, the TWGs reviewed the set of conditions prioritized for each life stage and reached consensus. Each team evaluated the list of conditions proposed for their respective age cohort and modified as necessary. Special considerations were given to conditions targeted for eradication (e.g., polio), elimination (Maternal and neonatal tetanus, neglected tropical diseases); conditions of specific concern to certain age cohorts, such as pregnancy/new-born care and epidemic prone disease such as Ebola and COVID-19. Any disagreement was resolved by weighing the evidence/data presented and technical justification made for inclusion/deletion. Sierra Leone deployed an extra level of consultation using online survey, where stakeholders reviewed conditions selected and provided inputs. As a result, the following set of conditions were selected as key priority for the age cohorts listed (See Table 2).

**Table 2:** Selected conditions to be addressed by EHCP

**Country**	**Age Groups**	**Selected number of Conditions**
**Sierra Leone**	Pregnancy and Reproductive health	16
	under 5	26
	Childhood 5–19	45
	Adults 20–49	53
	Adults 50–64	45
	Elderly 65 +	41
**Botswana**	Pregnancy and Newborn	39
	29–59 months	21
	Late childhood 6–13 years	28
	Adolescents and Youth;14–29 years	15
	Adult hood-29–65	17
	Elderly 65 +	15

### Development of EHCP interventions

Based on the list of conditions selected, a draft set of interventions were extracted from the WHO AFRO EHCP toolkit and refined as necessary. Each technical working group (TWG) was presented with interventions corresponding to their respective list of conditions, mapped by public health function and level of care. The facilitators compiled and presented the draft menu of interventions for selected conditions to each TWG. In areas where there was minimal or no information within the WHO AFRO toolkit, the teams referred to literature for selection of effective intervention.

### Rationalizing the EHCP

The TWGs were provided with a preliminary menu of interventions. The teams were tasked to review and appraise interventions for 1) technical comprehensiveness 2) contextualization to the country needs and 3) mapping interventions to the appropriate levels of service delivery in Botswana and Sierra Leone. All groups had a chair facilitating the discussion and appraisal process. Each day, the TWGs were able to review 6–10 conditions for their respective cohort.

The comprehensiveness of the interventions was reviewed and amended by the teams of expertise as necessary. The interventions were tailored to the Sierra Leonean or Botswana context and aspiration, with each team deciding ‘what’ and ‘where’ the interventions would be best delivered. Program managers and medical specialist in the TWGs customized interventions to align with country norms and strategies. For instance, the TWG responsible for 65 + age cohort highlighted local challenges faced by the elderly, in accessing and utilizing health care services. This resulted in amendments of interventions across the public health functions (eg. Proactive engagement against stigmatization, regular screening and medication review, introduction of home-based care, decentralization of rehabilitative services including assistive technology, spiritual support as a component of palliative care etc.).

### Harmonization and cross referencing

At the end of each day, plenary discussions were held for TWGs to present progress made and discuss approaches used in rationalizing the EHCP. Each team presented sample conditions and highlighted issues for consensus. For instance, some groups paid little attention to rehabilitative and palliative care interventions, which the plenary noted as critical components. Some of the curative and diagnostic services were better suited at secondary level for some teams, whereas others supported for their availability in primary levels of care.

### Key issues

#### Decision on the levels of service delivery

One of the key issues raised consistently in countries was concerning the levels of care. Within primary care level in Sierra Leone, there are currently different types of health facilities with varied degree of service provision capacities. The essential package has grouped these together and defined interventions that should be availed at the primary care level instead of the facility types that constitute this level. The future investment of the country is guided by the aspiration to deliver essential interventions to all ages, through incremental changes and upgrades of facilities, in order to shift current focus on certain age cohort (eg. facilities attaining to mother and child-care only) to phased expansion and access for all ages.

In Botswana, it was noted that the country has various policy documents that spell out different levels of service delivery. For example, some documents have included community level, health posts, outreach posts, health centers, primary hospitals, district hospitals, referral hospitals, tertiary hospitals while others, such as the 2010 package had community, clinics, primary hospital, district hospital and tertiary hospital as the levels. The team highlighted that Botswana also has a quaternary level facility. To bring consensus, the MOH team gave a presentation on the levels of service delivery, which the team was to utilize for the essential package. This process from both countries however indicates the ambiguity that exists in the categorization and the need for standardization.

## Discussion

The proceeding of country EHCP development have presented lessons learnt, core areas of focus for process and content changes. These are discussed and grouped under the following thematic areas.

### Conditions selection

Selection of conditions to be addressed by EHCP can benefit from utilization of different consultation modes and rich evidence on health emergencies. In order to maximize reach and stakeholder engagement early in the EHCP process, an online survey for prioritization of conditions by age cohort was deployed in Sierra Leone. This new approach adds a further step for wider consultation, in the initial stages of the EHCP. The survey provided an opportunity to add new conditions of public health concerns or of heightened importance to niche population. Although burden of diseases was utilized to determine morbidity, mortality and risk factors, conditions of public health importance were not as rigorously reviewed. The country experiences indicate that there is need to present data on potential shocks/outbreaks/epidemics as well as public health emergencies, as part of the preliminary review and evidence synthesis.

While global burden of disease estimates was utilized, the need to strengthen country health information systems was pronounced. In addition to global estimates, Botswana utilized estimates from its national statistics office. However, with the apparent gaps in health information and disaggregation by age groups, the country team agreed emphasis will be given to local systems to generate and access different types of data sources (facility, population data, surveillance etc.).

### Defining the levels of care for EHCP

The levels of care for EHCP delivery were frequently flagged out for consensus in countries. Experiences in Sierra Leone, Botswana, Eritrea, and Lesotho have indicated that different stakeholders and beneficiaries are likely to have diverging interpretation of the levels of care within their setting. The disparities in health facility types as well as levels of care in countries exemplify the gaps in service delivery platforms conceptualization and emphasize the importance of universal access. Provision of selected PHC services in different health facility types, within the primary level, is likely to exacerbate the already existing inequities to physical access [12, 13]. There is dire information and guidance on the optimal level of care structures within the African region and context, for universal access to efficient, effective, and quality services. In addition to regional definition and harmonization of optimal levels of care, it is recommended countries explicitly define the service delivery platforms prior to rationalizing their Essential health care packages.

### EHCP’s specificity

The level of EHCPs specificity, in terms of the interventions stated, was noted to vary across the technical working groups. This was more pronounced for the newly added interventions by countries, whereby the TWGs constructed the intervention statement, as opposed to customizing from the standard AFRO compendium. This is parallel to the findings in literature, whereby review of packages in low- and middle-income countries indicated the lack of harmonization on the EHCP specification [4]. In part, this is due to the heterogenous purpose of EHCP in countries, ranging from political instrument for resource mobilization to practical accountability means for service delivery aspirations. In some high-income countries such as United States of America, high level of EHCP specificity was deemed necessary based on scientific evidence, to explicitly indicate services included and excluded. On the contrast, others have argued that a balance is required between details and freedom for the health care workers to alter treatment and approaches as necessary, based on patient needs and national guidelines. The issue raised by countries necessitate regional guidance on development of services, in order to minimize discrepancies and outline optimal levels of specificities in EHCP.

A recent review of EHCPs in low- and middle-income countries outlined the variations that exist in the scope of interventions in countries against the World Bank’s Disease Control Priorities third edition (DCP3) [4]. Although 96% of the assessed EHCP samples covered RMNCAH and infectious disease interventions, non-communicable diseases were not as predominant. Palliative care and pain management, critical public health interventions, were only included in 19% of the packages. Furthermore, most packages do not explicitly state public health and clinical interventions, depicting the necessity to provide guidance on the content and process of EHCP rationalization in countries.

### Diagnostics as a public health function

Early diagnosis is proven to increase health outcomes and reduce out of pocket expenditure, a critical component for UHC attainment. However, diagnostics, including imaging are relatively weak links in the health systems of African countries [14]. Recent findings in availability of essential diagnostics indicated major gaps in primary care facilities (median of 19% for basic and 49% in advanced primary care facilities) in contrast to hospitals (68.4%) [15]. More importantly, significant variations were noted across conditions – with mean availability ranging from 1.2% (ultrasound) to 76.7% (malaria) in primary care facilities. Furthermore, COVID-19 has highlighted the fundamental role of diagnostics for effective health care management and national health security. Given the underrepresentation within the African region, rationalization of diagnostics in the EHCPs may be essential, for targeted focus and investments. Countries can opt to define their diagnostic services as part of their essential health care packages, to increase visibility and explicitly specify the set of diagnostic services at each level of care.

### Guidance on auxiliary pillars

The conceptualization of EHCP and its auxiliary pillars necessary for implementation remain largely undefined in the WHO AFRO region. Services delivery systems—the way the health sector organizes and manages the available investments – and service delivery outcomes, range of results arising from the health and health-related service interventions available to the population – are yet to be fully distilled in the context of WHO AFRO regional countries. This includes benefit packages (set of services country can afford currently), clinical guidelines and standard operating protocols, licensure and accreditation actions (focused on ensuring service provision units have the basic investment norms (licensure) and have continuous quality improvement initiatives (stepwise accreditation)); supervision and mentoring processes (aimed to ensure there is constant oversight and support to the care provision process); care standards and audits (defining the expected technical norms to be adhered to in provision of care, especially in ensuring person-centered standards); and patient safety initiatives (aimed to prevent and reduce risks, errors and harm that occur to beneficiaries during use of essential services) [9].

### Lifespan of the EHCP

EHCP is expected to guide countries’ aspirations for delivery of essential services. However, the timeframe is varied across the region, creating discrepancies and inconsistencies for stakeholders. The lifespan of the EHCP is partly varied in countries, due to the varied applications and designated purpose of the package. Countries have reiterated the necessity to align EHCPs with the national health policy timeframes, for tandem implementation with axillary pillars and health systems investments. Furthermore, alignment with the national policy caters for the long-term investments required to operationalize essential packages, such as health workforce, medicines, and health products, as well as infrastructure needs of the country.

### Monitoring of EHCP

EHCP implementation and monitoring was frequently raised as an area of priority. Regular monitoring can benefit countries in realigning strategic directions of health system priorities and investments. Some attempts are made to assess degree of EHCP availability to population, although substantial difference exist across and within countries, limiting analysis on coverage of interventions [16]. For instance, Malawi conducted sector wide approach (SWAp) impact evaluations through EHCP interventions availability and coverage [5], whilst other teams in the country explored distributional cost-effectiveness analysis of Malawi benefit package, focusing on health inequality [17]. Given the wide spectrum of EHCP uses and phased modalities of implementation, structured facility-based services assessment, such as the harmonized health facility assessment, could be leveraged for holistic review of services delivered. Furthermore, investments and regular monitoring of health systems functionality remains pivotal, as a strong predictor of UHC [18, 19].

### Way forward and follow up actions

The respective Ministries of health believe that the development of EHCP not only allows for more effective and equitable health service delivery, but also for the establishment of a functional referral system and allocation of appropriate investments for high impact interventions. The EHCP is expected to set precedence in defining ‘essential’ set of services for the population, structurally promoting integration of health services, and providing succinct guidance to partners and stakeholders on the country priorities. The process and outputs illustrated in this paper are expected to depict a novel and fit-for-purpose approach that can be deployed by countries.

**Fig. 2:**
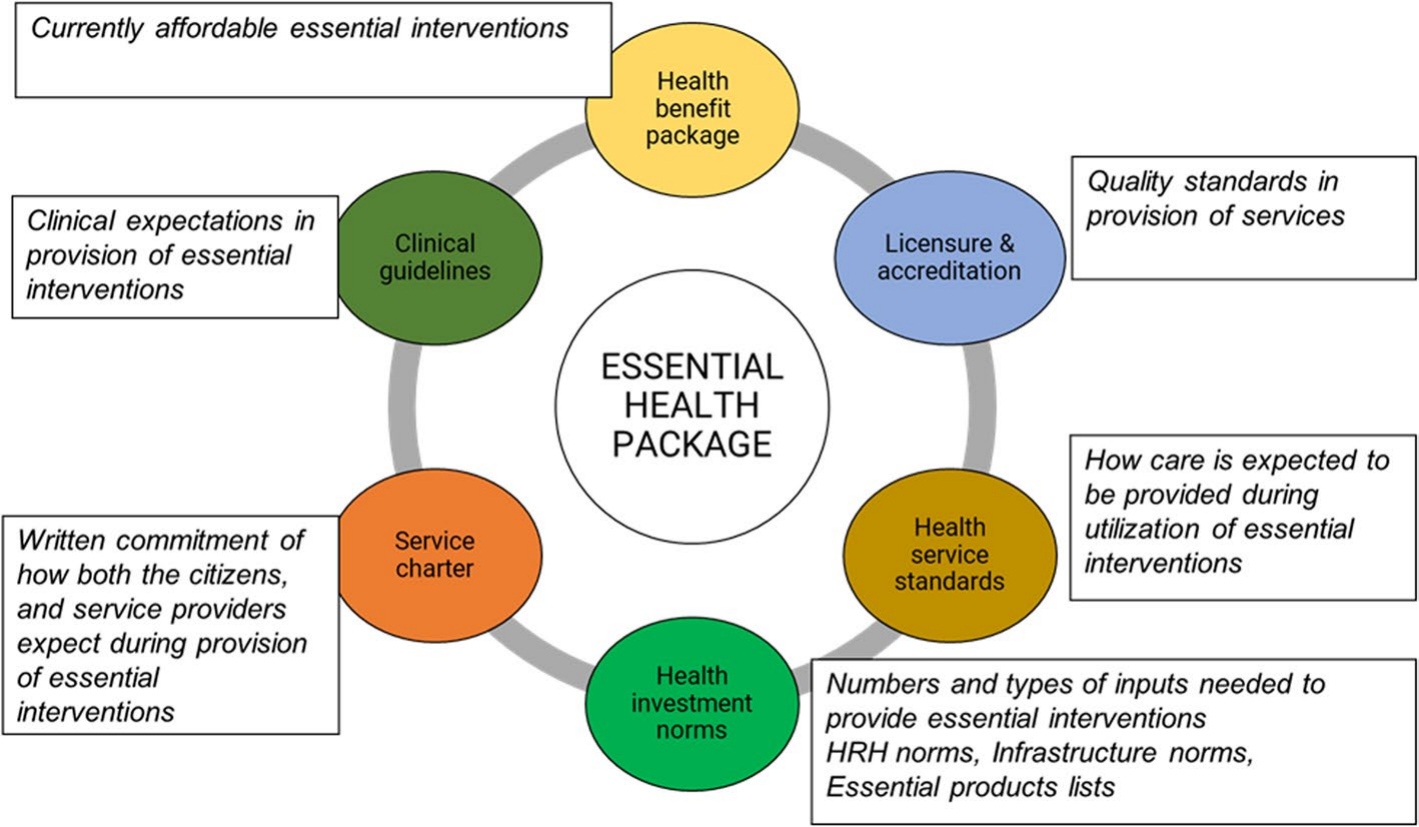
Operationalizing the EHCP

The Ministries of health and stakeholders are cognizant of the other critical arms needed for full operationalization of the package (Fig. 2). The conceptualization and proceedings of the EHCP development have outlined the subsequent auxiliary pillars required. Defining the package is a fundamental step for the health sector, which will subsequently guide the health investment norms (human resources, medicines, infrastructure etc.); delivery modalities (Service flow across the levels of care), health services standards (clinical guidelines, standard operating protocols); health benefit packages (currently affordable essential interventions); licensure and accreditation (adhering to the desired quality); and service charter (written commitment provided to general population on entitlements).

## Conclusion

The proceedings of Botswana and Sierra Leone are exemplar concepts of how member states can re-align investments and approaches for attainment of UHC through EHCP development. Similar to the instances observed in other African countries, the process promotes integration of services and shifts focus to the person, instead of a program or disease. Furthermore, it is worth noting that implementation of essential packages is highly reliant on engaging various health and health-related stakeholders, customizing interventions for beneficiaries based on their niche needs, managing risk factors early and investing in systems holistically, for the greatest impact. Many countries are striving to re-pivot their health systems, in order to meet the evolving contextual needs of the population and ensure their systems remain fit for purpose [19]. Thus, EHCPs can be utilized to guide health sectors inputs, for robust functionality of systems and attainment of UHC [17].

## Abbreviations

BoD: Burden of disease
COVID-19: Coronavirus disease 2019
DCP3: Disease Control Priorities third edition
EHCP: Essential health care package
iAHO: Integrated African Health Observatory
MDGs: Millennium Development Goals
MOH: Ministry of Health
RMNCAH: Reproductive Maternal Neonatal Child and Adolescent Health
SDGs: Sustainable Development Goals
SWAp: Sector wide approach
TWG: Technical working group
UHC: Universal Health Coverage
WHO AFRO: World Health Organization African Region
